# Mapping the field: A bibliometric literature review on technology mining

**DOI:** 10.1016/j.heliyon.2023.e23458

**Published:** 2023-12-14

**Authors:** Xinyue Hu, Huiming Gu, Yongli Tang, Bo Wang

**Affiliations:** School of Management, Jinan University, Guangzhou, China

**Keywords:** Technology mining, Bibliometrics, Co-citation analysis, Bibliographic coupling, Emerging topic

## Abstract

Technology mining (or tech mining, TM) is an emerging research field in science, technology, and innovation studies. However, due to the rapid increase and widespread application of TM research, accurately capturing research topics and emerging developments in TM has become a challenge for scholars. Therefore, this bibliometric literature review combines quantitative methods and content analysis to explore the research foundation and development frontiers of TM and distinguish emerging research topics from relatively mature ones, aiming to deepen the understanding. More specifically, it utilizes co-citation analysis and bibliographic coupling techniques to analyze the TM publication dataset. The results indicate that TM research is mainly based on four foundational areas, and there are five current frontier clusters. Emerging topic detection further shows that technology topic analysis, technology opportunity analysis, and technology management and decision support are currently emerging TM research topics.

## 1 Introduction

Porter [1,2] defined tech mining for the first time, that is, it mainly applies text mining tools and other methods to mine scientific, technological, and innovative information, to generate practical intelligence and support decision-making in areas such as technology monitoring, technology process management, and science and technology indicators. Since 2005, the number of scientific publications mentioning TM has gradually increased [3], and TM has garnered substantial interest as a text data-oriented analysis technique. In 2011, the first Global Technology Mining Conference was held in the United States and has since been held annually, providing an academic platform for researchers to share knowledge and exchange ideas. In recent years, the progress of text mining and other approaches and tools, as well as the application of TM in many fields, make TM one of the most promising research fields in innovation management [4].

Porter and his research team have long been paying attention to the development trends of TM. Their special issues on tech mining conferences provided useful insights into TM's current status and future research avenues. Chiavetta and Porter [5] summarized developing and implementing bibliometrics, text analysis, and visualization to obtain available information. Zhang et al. [6] pointed out that TM was developing in two directions: applying the approach of combining scientometrics and information technologies to analyze data, and building interdisciplinary networks of multiple fields to facilitate the application of TM. Huang et al. [7] elaborated on three aspects that need to be addressed in future TM: fully leveraging the wealth of conventional and new data, improving and/or integrating methods, and transforming innovative analysis into valuable intelligence.

On the other hand, due to the rapid increase and widespread application of tech mining research, accurately capturing development frontiers and emerging topics in TM has also become a challenge for scholars. Therefore, systematic and coherent analysis and reflection on technology mining research are necessary. Madani [3] used the CiteSpace bibliometric tool to quantitatively and qualitatively analyze 143 papers from the perspectives of authors, keywords, journals, universities, and countries, to review and prospect the development process and trends in the research field of tech mining. However, Madani's bibliometric analysis of TM was conducted in 2013, and there was no research foundation for analyzing TM. After 2013, the number of tech mining papers has grown rapidly (see section 3.1 for relevant evidence). Analyzing the literature on TM in the past decade helps us better grasp the frontiers and emerging developments of TM, and exploring the research foundation is conducive to our comprehensive understanding of TM.

Consequently, this article adopts co-citation analysis, bibliographic coupling, and emerging research topic detection to conduct a bibliometric literature review on tech mining, aiming to explore the research foundation and development frontiers of TM and identify emerging TM research topics. The basic idea of the two citation-based methods is to measure the thematic similarity between scientific publications through the overlap of citation patterns. Co-citation analysis can be used to trace the scientific roots of the research field, while bibliographic coupling can be adopted to identify current research topics and future research trends [8,9]. This article utilizes these two bibliometric techniques to capture the scientific foundation and the development frontier of TM research. Moreover, identifying emerging research topics in a research field is particularly beneficial for research foundations and policymakers, as it can promote and strengthen the development of potentially promising research topics [10]. Therefore, based on the cutting-edge development of TM, we detect and identify emerging TM research topics.

This paper analyzes 277 peer-reviewed articles related to tech mining, supplementing and cross-validating earlier research findings. The results of the co-citation analysis show that the research basis of TM includes four aspects: (1) text analysis of emerging technology, (2) bibliometrics, (3) patent analysis, and (4) strategic technology management. In turn, the results of bibliographic coupling indicate five current and frontier thematic clusters: (1) technology topic analysis, (2) technology roadmapping, (3) technology component analysis, (4) technology opportunity analysis, and (5) technology management and decision support. Among them, technology topic analysis, technology opportunity analysis, and technology management and decision support are emerging research topics.

This article makes the following contributions: firstly, this study applies co-citation analysis and bibliographic coupling to explore the field of tech mining research, revealing the research foundation and evolution process of TM, and showcasing the development frontiers of existing research, and comprehensively reflecting the research field of TM. Secondly, we evaluated the relative importance of research themes and detect and identify emerging research topics to drive future research and development in TM. Finally, through content analysis of the development frontiers and emerging topics, this study proposes several future research directions.

The rest of this paper is structured as follows. In the next section, we provide a detailed introduction to the data, methods, and analytical tools used in the bibliometric review. In the third part, we elaborate on the main results of co-citation analysis and bibliographic coupling and identify emerging research topics. In the fourth part, this article discusses the implications and contributions of the research findings. Finally, the limitations of this study are discussed and future research directions are proposed.

## 2 Methodological approach for the bibliometric literature review

### 2.1 Bibliometric literature review steps

**Fig. 1:**
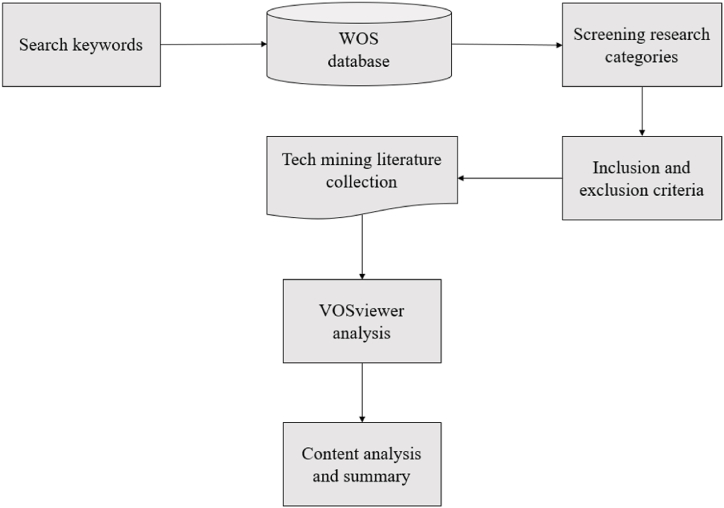
Overall research framework.

To identify the research foundation, development frontiers, and emerging topics of the tech mining field, this article conducts a bibliometric literature review study combining specific methods. The research framework of this paper is shown in Fig. 1. A bibliometric review answers a series of research questions by systematically reviewing and analyzing all published articles within a specific scope. The bibliometric review of this article includes the following steps: (1) determining the citation database and retrieval time, (2) determining search keywords, (3) screening research categories, (4) constructing inclusion and exclusion criteria, (5) manually coding the literature collection, (6) clustering and content analysis, and (7) identifying emerging topics.

Step 1 determining the citation database and retrieval time

This paper retrieved the target article from the SCIE and SSCI citation index sections of the Thomson Reuters Web of Science database (WOS). The WOS database is well-known for its comprehensive coverage of relevant journals and high-quality peer-reviewed publications. We searched for the literature of article type published between 2006 and 2023. The reason for setting the search start year to 2006 in this article is as follows: Porter [1] first defined the concept of ‘technology mining’ in his book, which was published in 2005. This was the first time that the academic community defined such research content as ‘technology mining’, which was then widely accepted by researchers. Although similar work has been done to achieve similar goals in previous studies, with the support of this definition, research on technology mining has become clearer and more standardized. The literature used for analysis in this article was retrieved on January 12, 2023. Therefore, 2023 is the end year of the search, but the data for 2023 is incomplete and cannot be used as a total value for the entire year.

Step 2 determining search keywords

The topic search constructed in this paper drew on Madani's tech mining search query logic [3], and modified and improved the retrieval content based on the concept of technology forecasting [11], author keywords, and keyword plus assigned by WOS. In addition, considering that the keywords used in TM methods were constantly evolving, this article appropriately expanded the search content of this part. Based on these, the final topic search has been constructed (see Appendix).

Step 3 screening research categories

Due to the focus of this article on the application of tech mining in ST&I, to ensure the relevance of search results, we screened the research categories of the literature. This article examined the research areas of S&T research identified by Martin et al. [12] and the WOS research category selected by Ranaei et al. [13]. We identified 9 research areas that are consistent with the latter, including Business, Management, Information Science Library Science, Computer Science Interdisciplinary Applications, Regional Urban Planning, Operations Research Management Science, Multidisciplinary Sciences, Economics, and Social Sciences Interdisciplinary.

Step 4 constructing inclusion and exclusion criteria

Although the research category screening process was conducted, to further improve the accuracy and relevance of the literature, after repeated discussions within the research team, we proposed the inclusion and exclusion criteria for selecting articles. To establish the final literature sample, this research posed six questions based on the definition of TM as criteria for including and excluding publications. These issues are summarized as follows:

1. Does the study address tech mining methods and applications as a main inquiry?
2. Does the study concern ST&I information analysis based on large-scale data (e.g., patents, publications, social media, and other online sources)?
3. Does the study mainly aim at technology management purposes (e.g., tech mining purposes in search query …)?
4. Does the study use analytics tools for tech mining (e.g., tech mining tools, bibliometrics, machine learning, data mining, text mining, natural language processing, network analysis …)?
5. Does the study mainly concern the development of new or improvement of existing tech mining methods and procedures?
6. Does the study mainly concern the application of tech mining methods and procedures for obtaining technical intelligence?

If the response to question 1 and any of the following questions (2, 3, 4, 5, or 6) is positive (i.e., Yes), an article is included in the final literature set.

Firstly, two team members independently screened the initial literature collection, carefully read the title, abstract, and keywords, and divided the article into three categories based on inclusion and exclusion criteria: inclusion, exclusion, and uncertainty. Then, based on the screening results obtained by each of the two team members, a small amount of literature with inconsistent screening results was read, discussed, and classified. Next, downloaded the uncertain literature and read the full text, and discussed them with the third team member. Finally, we obtained a refined collection of 277 articles on technology mining.

Step 5 manually code the literature collection

After obtaining the refined TM literature set, we exploited manual analysis to complete the manual coding of the literature set and gain a rough understanding of the overview of TM. Two research members independently read 277 papers, and after discussion, standardized coding schemes were used to code the literature collection. We drew on the experience of Antons et al. [14] to extract the basic information, data type, and time of each article, and subdivide the types of text mining methods and the main purpose of TM. The manual coding results of the literature collection can be found in Supplementary Material.

Step 6 clustering and content analysis

We utilized the co-citation analysis and bibliographic coupling functions in VOSviewer software to cluster relevant literature on TM. Through content analysis, we elaborated on the clustering results to identify the research foundation and development frontiers of TM.

Step 7 identifying emerging research topics

Finally, this article distinguished between emerging and relatively mature topics in cutting-edge research on TM, providing a theoretical basis for subsequent analysis and recommendations.

### 2.2 Methods and visualization

To analyze the mapping model of articles in the literature set and evaluate the similarity of tech mining literature themes, two bibliometric techniques, co-citation analysis, and bibliographic coupling, are applied [[15], [16], [17]]. The reason why these two techniques can effectively evaluate the thematic similarity of scientific publications is that they rely on a fundamental assumption that there is a certain degree of relatedness between a publication and its references [8]. Co-citation analysis can be used to study the origin and basic theories of a certain field; Bibliographic coupling is more suitable for exploring the present research status and trends in a certain field [15]. Therefore, based on the properties of the two, this article applies co-citation analysis to identify the research foundation of TM and uses bibliographic coupling to identify the development frontiers of TM.

The visualization of similarities (VOS) mapping technique proposed by Van Eck and Waltman [18], as well as the VOSviewer program, are used in this article to assess the relatedness of articles in the tech mining literature collection to discover and depict research themes. This article performed a content analysis on publications of various clusters according to the clustering outcomes. We labeled and summarized the main content of each cluster through term frequency analysis, combined with the titles, abstracts, and keywords of clustered publications. To provide constructive research suggestions, this article, combined with previous manual coding work, implemented a thorough reading and qualitative analysis of the 10 articles with the highest citation quantity in each cluster. Moreover, the research team read additional relevant literature while exploring future research directions and practical applications of tech mining to ensure the richness of tech mining research.

Although previous studies have developed many indicators, the definition and attributes of emerging topics have been ambiguous for a long time, and the linkage between concepts and created indicators is incomplete, resulting in these indicators failing to efficiently identify emerging research topics. Wang has defined emerging research topics based on the definition of emerging technologies [19] and proposed effective measure criteria to fill the existing gap [20]. The emerging research topic is a very novel and rapidly growing research topic, with a certain degree of coherence and significant scientific impact. Growth, novelty, scientific impact, and coherence are four attributes of an emerging research topic [20]. This article drew on Wang's experience, we denote $p_{i,t}$ as the number of publications on the topic i in year t, $p_{i,t+\Delta t}$ as the number of publications in year t+Δt, where Δt represents the time interval. So, the growth rate of a topic i in the interval Δt can be defined as

(1) $$r_{i,t+\Delta t}=p_{i,t+\Delta t}/p_{i,t}$$

However, the number of publications is often susceptible to random fluctuations, which would cause a sudden increase or decrease of yearly publications. To alleviate this situation, we use the smoothed annual number of publications instead of the actual number of publications following [20]. This is obtained by calculating the average number of publications in three consecutive years, which can be expressed as

(2) $${\bar{p}}_{i,t}=\left(p_{i,t-2}+p_{i,t-1}+p_{i,t}\right)/3$$

Correspondingly, the growth rate equation (1) can be revised by the following formula

(3) $$r_{i,t+\Delta t}={\bar{p}}_{i,t+\Delta t}/{\bar{p}}_{i,t}$$

Novelty indicates that the number of publications on a topic should be relatively small in the early stages of emergence, that is, ${\bar{p}}_{i,t}$ should be relatively small. Moreover, citations are considered indicators of whether emerging topics have an outstanding scientific impact and $c_{i,t+\Delta t}$ represents the number of citations to publications published between t and t+Δt in topic i. Similarly, emerging topics should be coherent. If the total number of citations in a given cluster is less than the total number of publications, then the publications within the cluster may be loose. The coherence of a topic can be measured by dividing the total number of citations by the total number of publications, denoted as $h_{i}$. If a topic meets the above criteria, it is an emerging research topic.

## 3 Results

### 3.1 Descriptive results

**Fig. 2:**
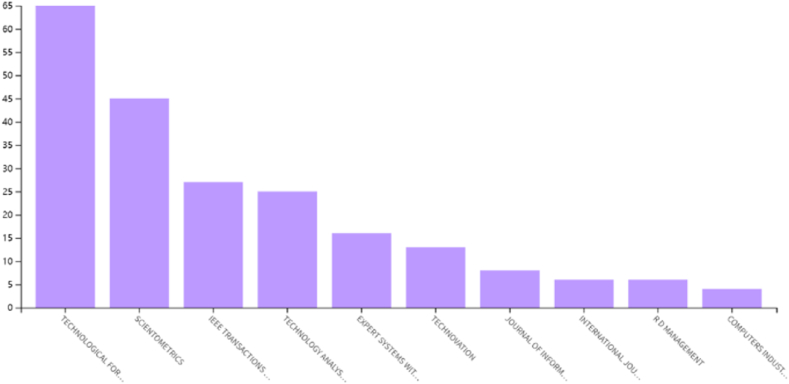
Distribution of source publication names.

**Fig. 3:**
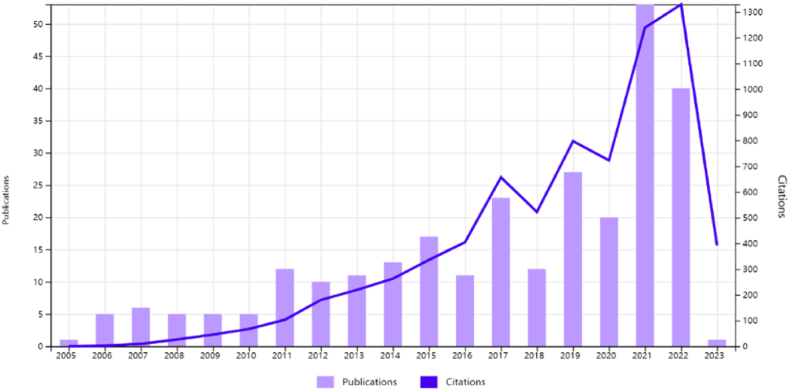
Citation frequency and publication distribution by year.

This article provided a brief analysis of 277 tech mining literature using WOS analysis tools. These papers have 3873 citing articles, with 7304 times cited, with an average of 26 citations per paper. The H-index is 47, indicating that 47 articles have been cited at least 47 times, and the selected papers have a significant impact. In addition, the ranking of publications is shown in Fig. 2, with the top three journals being Technological Forecasting And Social Change (65), Scientometrics (45), and IEEE Transactions On Engineering Management (27), accounting for half of the 277 papers. Fig. 3 shows the citation frequency and publication distribution of 277 papers, indicating that tech mining research is generally in a rapid development stage. Especially since 2013, the number of publications has significantly increased, and the citation frequency curve is significantly steep, indicating that the field of TM research has received increasing attention in the past decade, and more researchers have begun to invest in this research work. This on the other hand confirms the necessity of conducting a new round of literature review.

### 3.2 The foundations of tech mining research

**Fig. 4:**
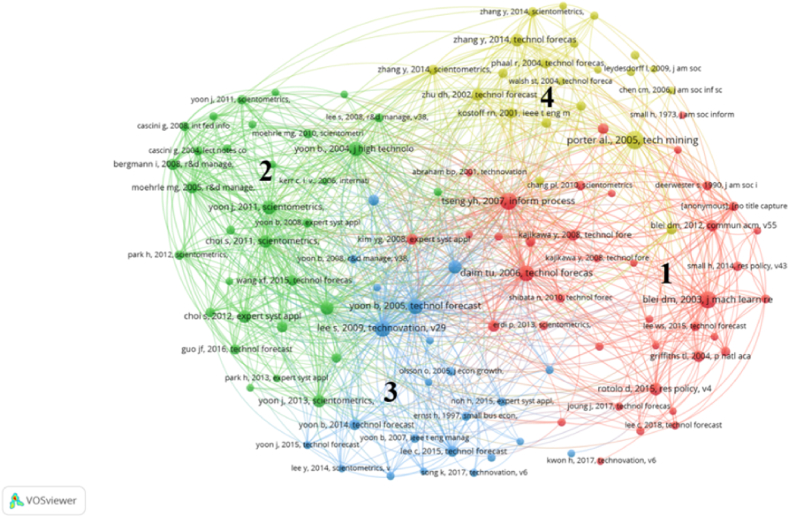
Co-citation network of the references cited by publications on tech mining.

To analyze the research foundation of tech mining, we used a co-citation analysis on the literature collection, and the results are shown in Fig. 4. The literature collection of this article cites a total of 10285 references. To make the selected cited references have a certain influence, the minimum citation threshold for each cited reference is set to 8, resulting in a total of 175 cited references being selected. Among them, two papers are the same, and we merged them using the VOSviewer software. However, due to our main focus being to explore the foundation of tech mining research beyond the literature set itself, we excluded 50 articles that belong to both the cited reference set and the tech mining literature set [8]. Moreover, three records cannot assist us in accurately retrieving cited references, and we considered these publications as missing observations. In the end, we obtained 121 references.

From Fig. 4, it can be seen that the references are divided into four clusters. To explain and label the clustering content, we downloaded the 121 cited reference records, read the titles, abstracts, and introductions of the cited literature, and listed the most common terms in the titles, abstracts, and keyword sections of each clustering reference.^1^ Based on this effort, we labeled these clusters as ‘Cluster Red (1) – Text Analysis of Emerging Technology’, ‘Cluster Green (2) – Bibliometrics’, ‘Cluster Blue (3) – Patent Analysis’, and ‘Cluster Yellow (4) – Organizational Strategic Management’. In Fig. 4, each circular icon represents a reference, and the larger the area of the circular icon, the more times the reference has been cited. Moreover, the distance between a pair of references represents the likelihood of being co-referenced, and the closer the distance, the greater the likelihood. Next, we will describe and discuss the research content of each cluster. Although this article attempts to reveal the overall appearance of each cluster, we need to acknowledge that our analysis cannot fully elucidate the richness of each cluster.

#### 3.2.1 Cluster 1: text analysis of emerging technology

The research on this clustering mainly introduces text analysis techniques applied in emerging technological fields. Text analysis is a basic problem of text mining, which can quantify the features extracted from the text to represent the text information. Therefore, the text analysis technique is crucial for understanding and obtaining useful technological intelligence on emerging technologies. The cluster article mainly applies topic modeling methods to study and analyze text data of emerging technologies. Topic modeling is a method mainly used to infer the probability distribution of text topics, which can effectively identify scientific topics [21,22]. At present, patent engineers or decision-makers have a great demand for automated text analysis tools of emerging technologies [23]. For example, scholars utilized methods such as LDA topic modeling to extract topics from emerging technologies and combined them with time dimension to identify hot topics [24]. Further research proposes an advanced research framework based on topic analysis to assist text analysis, such as using topic modeling to predict technological convergence [25], and identifying emerging topics in science and technology [26]. Moreover, the cluster article also focuses on other text analysis techniques, including text visualization, latent semantic analysis, and text similarity measurement [[27], [28], [29]], aiming to help managers understand the information and structure of emerging technologies and predict technological development trends.

#### 3.2.2 Cluster 2: bibliometrics

The main research contents of this cluster are tech mining and bibliometrics. After Porter [1] proposed the concept of technology mining, obtaining ST&I information has increasingly attracted the attention of scholars and practitioners. Accordingly, research belonging to this cluster focuses on the use of bibliometrics to analyze patent documents and scientific publications to understand how emerging technologies are discovered, evaluated, and utilized, and what impact they have. In this cluster, some papers have conducted technological forecast research through bibliometrics. For example, bibliometrics was combined with other technical analysis tools to predict the development dynamic of emerging technologies [30], and the bibliometric indicators of innovation models were constructed to predict the development trend of technologies [31]. There are also some studies on improving the tech mining preprocess through bibliometrics, including improving the retrieval strategy [32], and improving the analysis effect of data [33]. In addition, some articles use bibliometrics to excavate technology information for generating cartography of technology. For instance, bibliometrics was combined with qualitative methods and visualization technology to build a technology roadmapping [[34], [35], [36]], aiming to track the trajectory of technological development and explore the time-varying relationship between technologies.

#### 3.2.3 Cluster 3: patent analysis

The core content of this cluster is to leverage text mining methods to scour patent information for meaningful technological intelligence. Among them, citation analysis can find the relationship between patents, and then reveal the relationship and development law between technologies. Scholars reported a direct validation study of patent citation analysis and found that there is a strong association between the number of citation counts and the importance of patents and technology [37]. As a result, citation analysis has received extensive attention in patent analysis. However, some scholars noticed some shortcomings in citation analysis, and then proposed patent citation network analysis and some related indicators to quantitatively analyze patent information [38]. Afterward, some researchers applied patent citation networks to represent patent connectivity relationships to analyze and predict technological development and diffusion [39,40]. On the other hand, semantic analysis can also effectively analyze patent data. Subject-action-object (SAO) semantic analysis focuses on the property and function of patents, which can be used to identify the relationships between technologies. Some studies have utilized SAO semantic analysis to improve the organization's patent analysis capabilities [[41], [42], [43]]. In addition, some scholars have paid attention to semantic TRIZ (Theory of Inventive Problem Solving), which can assist managers in identifying technological potential and future development directions [[44], [45], [46]].

#### 3.2.4 Cluster 4: strategic technology management

The literature in this cluster focuses on using tech mining to analyze data to fulfill organizational strategic technology management goals. The core elements of this cluster are to manage company technology knowledge, identify enterprise technology opportunities, and predict industry technology trends by scouring technical information. Some studies analyzed patent information to identify and evaluate potential sources of externally generated technical knowledge [47], which promotes the management of technological knowledge in companies. Technology opportunities are one of the fundamental determinants of technological progress in the industry. Effective and efficient identification of technology opportunities enables managers to maximize the utilization of existing technology, thereby helping them formulate technological strategies [[48], [49], [50], [51]]. For example, scholars have proposed an improved morphology analysis method that captures technical morphological information that can support strategic planning for internal research and development (R&D) or licensing within the organization [51]. Besides, technology forecast is also the “catalyst” for strategic technology management [52,53]. The latter adopted patent clustering and technology life cycle to predict the development process and trend of technology. Finally, the cluster also includes other strategic technology management content, consisting of detecting technology vacuum [54,55], and monitoring technological convergence [56].

#### 3.2.5 The relative importance of clusters of cited references

To measure the relative importance of each cluster and analyze their impact on tech mining research, we calculated relevant statistical data for each cluster based on the number of publications, age of existence, and number of citations. We also listed the three most cited articles in each cluster. It should be noted that the citation quantity of references in the statistical data is the number of references cited by the technical mining literature set, rather than the total number of historical citations. These statistical data are shown in Table 1. The fifth column of the table shows the average age of references, while the sixth column provides the average citation count of each reference. Cluster 1 (text analysis of emerging technology) occupies a dominant position in terms of the total number of publications and citations, indicating that scholars have a great interest in the research content of cluster 1. Although the number of publications of cluster 2 (bibliometrics) is slightly less than that of cluster 1, the average number of citations shows that the seminal works in the tech mining research area it covers are more influential. On average, the references in cluster 2 were cited the most in the tech mining literature collection (15.1 times), while the average citations belonging to cluster 3 (patent analysis) and cluster 4 (strategic technology management) were 12.2 and 12.8 times, respectively. In addition, only the literature belonging to clusters 1 and 2 was cited more frequently than the average citation value (13.9). Meanwhile, cluster 1 is the youngest cluster to exist in the year of publication, with an average of 14.9 years, indicating significant development in patent analysis research in the past. Cluster 3 (patent analysis) has a long history of evolution (20.5 years), which has greatly shaped the development model of tech mining research and provided a solid foundation for current and future research progress.

**Table 1:** Key statistical indicators of the clustered publications, based on co-citation analysis.

Cluster	Cluster label	Number of publications	Total number of citations	Average age of publications (in years)	Average number of citations per publication	Three most cited articles; citations
1	Text Analysis of Emerging Technology	38	571	14.89	15.03	[22]; 53 [23]; 52 [19]; 28
2	Bibliometrics	32	482	16.75	15.06	[1]; 60 [30]; 45 [57]; 27
3	Patent Analysis	29	355	20.45	12.24	[38]; 38 [43]; 25 [42]; 19
4	Strategic Technology Management	25	319	16.48	12.76	[51]; 41 [58]; 33 [47]; 23
Total	N/A	124	1727	17.02	13.93	N/A

### 3.3 Thematic areas in tech mining

**Fig. 5:**
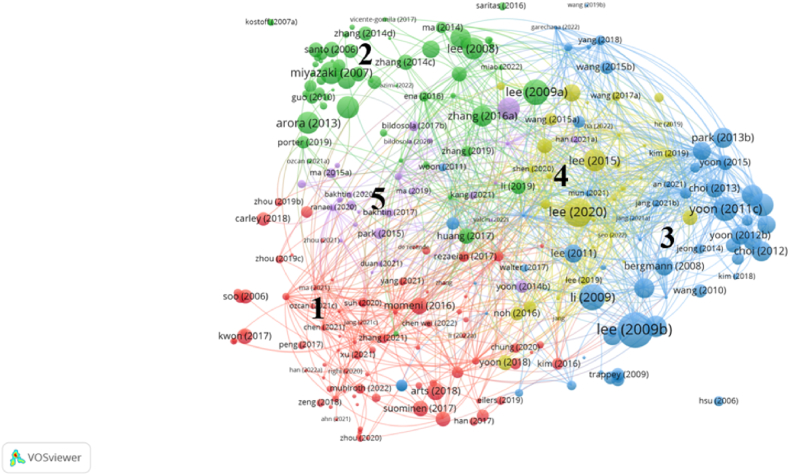
Bibliographic network of science publications on TM based on bibliographic coupling.

To analyze the current research status and emerging themes of tech mining, we conducted bibliographic coupling, and the results are shown in Fig. 5. Fig. 5 shows a relatively coherent bibliographic network, where we divided 277 publications into five clusters. To explain and label the cluster content, we adopted the same analysis method^2^ as co-citation research. Based on this effort, we labeled these clusters as ‘Cluster Red (1) – Technology Topic Analysis’, ‘Cluster Green (2) – Technology Roadmapping’, ‘Cluster Blue (3) – Technology Component Analysis’, ‘Cluster Yellow (4) – Technology Opportunity Analysis’ and ‘Cluster Purple (5) – Technology Management and Decision Support’. It should be noted that in Fig. 5, the area size of the circular icon represents the total link strength of each publication. Next, we will describe and discuss the research content of each cluster. Although this article attempts to reflect the full range of each cluster, we need to acknowledge once again that our analysis cannot fully elucidate the universality of each cluster.

#### 3.3.1 Cluster 1: technology topic analysis

In terms of the articles included the largest cluster focuses on utilizing tech mining methods to research technology topics. The article mainly focuses on applying LDA topic modeling, and sentiment analysis methods to analyze technology topics to promote organizational R&D progress and technological development. In this cluster, a large number of academics have used LDA topic modeling to identify potential technology topics in patent data. Further analysis based on technology topics can reveal technological development opportunities or threats [[59], [60], [61]]. In fact, scholars also used sentiment analysis methods when exploring thematic intelligence [[62], [63], [64]]. For example, scholars exploited these two methods to identify and select topics related to future society and future risks, respectively, to identify safe technological opportunities [65]. Moreover, researchers have also combined other methods and theories, such as SAO semantic analysis, machine learning, bibliometrics, technology life cycle, and expert judgment to investigate technology topics [60,66,67]. Accordingly, the methods proposed in these articles can effectively identify technology topics and potential development opportunities. These systematic methods have been applied in emerging technology fields such as Dye-sensitized solar cells, artificial intelligence, blockchain, etc. Furthermore, academics have analyzed the evolution of technology topics in patents and scientific research to obtain other technological intelligence, such as stimulating organizational R&D, evaluating technology competitiveness, and assessing project feasibility [[68], [69], [70]].

#### 3.3.2 Cluster 2: technology roadmapping

The focus of this cluster is to build a Technology roadmapping and forecast technology development trends to support the organization's planning and strategy formulation. This cluster article uses text mining technology, combined with network analysis, bibliometrics, and expert judgment, to generate a technology roadmapping to reveal the evolution process of technology in a certain field, understand the development trajectory of technology, and predict and identify promising technologies. The main research content is to develop a technology roadmapping research framework combining qualitative and quantitative analysis to visualize the technology evolution path and predict the technology development trend [[71], [72], [73]]. Specifically, scholars used technology-relationship-technology semantic analysis to extract the patent structure relationship and build a technology roadmapping from technology to market [74]; Academics have built a data-driven technology roadmapping combining topic modeling and link prediction, to insight and track the time-varying evolution path of technology [75]. In addition, considering the uncertainty factors in the future technology planning stage, some studies expect to combine other analysis approaches and tools, such as fuzzy cognitive maps [76], data-driven computer decision support systems [77], bayesian networks [78], to analyze the causal relationship between technologies, and propose a technology roadmap that can adapt to dynamic and complex environments and semi-automatic decision-making to accurately forecast technology development trends.

#### 3.3.3 Cluster 3: technology component analysis

This cluster deploys tech mining methods to analyze and identify technology components to obtain useful technological intelligence. Scholars mainly analyze patent data and technological documents through SAO semantic analysis, TRIZ theory (Theory of Inventive Problem Solving), network analysis, and expert suggestions to explore the properties, functions, and structures among technologies, and identify core technology components. This cluster focuses on utilizing SAO semantic analysis for tech mining, and the SAO structure can represent clear relationships between components in patents [79]. These researches clearly describe the detailed information between technology components in patents through SAO analysis, extract and screen the structural relationships of technology components, and then determine the core technology components, which can be used to monitor and predict the correlation and evolution between technologies [[80], [81], [82]]. Moreover, some studies explore the property and function of patents through semantic analysis and then exploit TRIZ trends for technical analysis to predict technological innovation and development directions [[83], [84], [85]]. For example, experts applied the SAO method to analyze patent data and used TRIZ evolution trends as a standard for evaluating patents, to identify high-value technologies [86]. Technology component analysis helps identify key and core technologies, and funding these technologies will promote rapid regional economic development. Consequently, understanding and monitoring the important technology components of technology is a key research issue that needs to be addressed in the field of technology management [87].

#### 3.3.4 Cluster 4: technology opportunity analysis

This cluster is mainly composed of articles that leverage methods such as morphology analysis, SAO semantic analysis, and local outlier factor analysis to identify technological opportunities. Here, the research focus is to employ systematic text mining methods to scan the technological environment to find potential and new technological opportunities. Morphology analysis can effectively list the possible technical combinations of all product components, among which feasible technical combinations are opportunities to discover and develop new technological solutions. Therefore, tech mining methods combined with morphology analysis are often applied in research on technology opportunity analysis [[88], [89], [90]]. On the other hand, researchers apply SAO semantic analysis and other methods to extract and analyze technological information from patent data, understand technological trends, and identify technological opportunities [[91], [92], [93]]. In addition, scholars measure the novelty of technological innovation at a numerical scale based on local outlier factors and identify new technological opportunities [[94], [95], [96]]. At a broader level, the article in this cluster uses methods such as link prediction, F-term, and oriented projected cluster generation algorithm to analyze and explore the technological opportunities of enterprises [97,98]. Furthermore, some studies aim to identify potential innovation paths of technology [99], identify potential target markets for products [100], and designate new research and development strategies [101]. It is not difficult to see that the literature in this cluster discusses various tech mining methods pursued at discovering technological opportunities.

#### 3.3.5 Cluster 5: technology management and decision support

The core content of this cluster is to analyze various activities of organizations in the process of technology research and development through text mining and other approaches and tools and to obtain the necessary experience, knowledge, and information to assist managers in solving technology management and decision support problems. Specifically, the research of the cluster mainly deploys bibliometrics, SAO semantic analysis, link prediction, and other methods to develop the analysis and research framework of scientific publications and patents. By utilizing these methods and tools, organizations are allowed to obtain rich technological intelligence, identify and select R&D partners that meet their needs, providing a theoretical basis for managers committed to promoting cooperation between research institutions and/or high-tech enterprises [[102], [103], [104]]. Besides, some articles in this cluster analyze and evaluate mature or emerging scientific and technological fields, identify technological development prospects, and provide valuable references for management and decision support in technology R&D activities [[105], [106], [107]]. Other studies on technology management and decision support use text mining to assess the level of technology development and predict the trend of technology development, to support managers to identify promising technology fields and study the structural mechanism of knowledge convergence [[108], [109], [110]].

#### 3.3.6 The development and impact of clusters over time

In order to evaluate the relative importance of bibliographic coupling themes, similar to section 3.2.5, we calculated some citation-based statistical data, as shown in Table 2. Firstly, from the fifth column of the table, it can be observed that, on average, publications belonging to clusters 2 (technology roadmapping) and 3 (technology component analysis) have a relatively long history in the field of tech mining. The average publication ages of publications belonging to these clusters are 8.5 and 8.1 years, respectively, which are higher than the sample average of 5.8 years. Clusters 3, 2, and 1 (technology topic analysis) are the three clusters with the highest number of citations among all clusters (see column 4), and cluster 1 is the ‘youngest’ publication cluster. In addition, the two clusters with the highest average number of citations per publication are 3 and 2, respectively 42.7 and 32.1, which are much higher than the total average number of citations (see column 6). In fact, if we control the age of publications, publications from cluster 4 (technology opportunity analysis) are the most cited, receiving an average of 6.5 citations per year. The annual citation count of publications in cluster 3 is also higher than the sample average. However, the average citation rate of publications belonging to clusters 1, 2, and 5 (technology management and decision support) per year is lower than the average level (see column 7). In summary, publications that focus on Technology Opportunity Analysis and Technology Component Analysis have higher citation influence.

**Table 2:** Key statistical indicators of the clustered publications, based on bibliographic coupling.

Cluster	Cluster label	Number of publications	Total number of citations	Average age of publications (in years)	Average number of citations per publication	Average number of citations per publication per year	Three most cited articles; citations
1	Technology Topic Analysis	87	1189	3.50	13.67	3.95	[111]; 71 [112]; 65 [113]; 54
2	Technology Roadmapping	63	2020	8.46	32.06	3.79	[72]; 150 [114]; 117 [71]; 114
3	Technology Component Analysis	60	2564	8.12	42.73	5.26	[115]; 279 [116]; 151 [79]; 135
4	Technology Opportunity Analysis	35	812	3.57	23.20	6.50	[100]; 190 [96]; 110 [89]; 68
5	Technology Management and Decision Support	32	422	4.86	13.19	2.71	[117]; 111 [104]; 37 [103]; 32
Total	N/A	277	7007	5.78	25.30	4.37	N/A

**Fig. 6:**
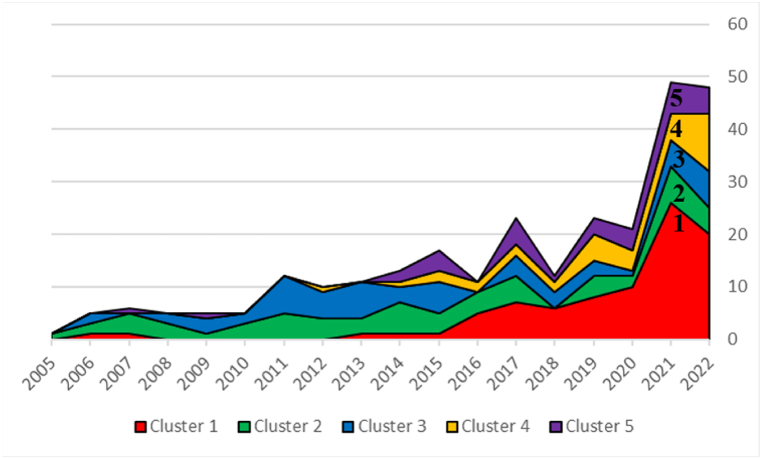
Distribution of the number of published papers per cluster per year.

We also plotted the distribution of the number of published papers per year for each cluster to evaluate the changes in the theme development of tech mining research over time. The curve is shown in Fig. 6. From the graph, it can be seen that the distribution of publications in the field of tech mining presents a ‘double prosperity’ structure, with publication production reaching its peak in 2017 and 2021. From the graph, it can be clearly seen that clusters 2 (technology roadmapping) and 3 (technology component analysis) have relevant research content almost every year during the investigation period. The research in clusters 1 (technology topic analysis), 4 (technology opportunity analysis), and 5 (technology management and decision support) only began to emerge after 2013, and the proportion of publication output in the past four years (2019–2022) is high, indicating that they have received increasing attention in recent years. In a nutshell, there has been no significant change in the theme orientation of clusters 2 (technology roadmapping) and 3 (technology component analysis) in the past 18 years; The research topic of clusters 1 (technology topic analysis), 4 (technology opportunity analysis), and 5 (technology management and decision support) has become increasingly important in recent years, especially technology topic analysis.

#### 3.3.7 Identifying emerging research topics

The main research content of this subsection is to detect and identify emerging research topics and relatively mature topics in the development frontiers of TM. The emergence attributes of each research topic are obtained through the following methods. We first determine the maximum growth rate for each topic at a given time interval. As described in section 2.2, the growth rate of a topic i in the interval Δt can be defined as

$$r_{i,t+\Delta t}={\bar{p}}_{i,t+\Delta t}/{\bar{p}}_{i,t}$$

${\bar{p}}_{i,t}$ is the smoothed number of publications on the topic i in year t, ${\bar{p}}_{i,t+\Delta t}$ is the smoothed number of publications in year t+Δt, where Δt represents the time interval. Based on this, the smoothed number of publications in the first year of the period and the number of citations during that period can be calculated. The coherence indicator can be measured by dividing the number of citations within a topic by the number of publications. In this way, we have gained the growth, novelty, scientific influence, and coherence of each research topic. Table 3 lists the emergence attributes for each research topic.

**Table 3:** Statistics on the TM-related research topics.

	Time interval	Total no. of publications	Begin year	End year	Novelty (${\bar{p}}_{i,t}$)	Growth rate ($r_{i,t+\Delta t}$)	Coherence ($h_{i}$)	Scientific impact ($c_{i,t+\Delta t}$)
Cluster 1	Δt=2	87	2015	2017	1	433 %	13.67	386
	Δt=5		2013	2018	0.33	1800 %		588
Cluster 2	Δt=2	63	2020	2022	2	233 %	32.06	88
	Δt=5		2010	2015	2.33	186 %		790
Cluster 3	Δt=2	60	2007	2009	0.67	250 %	42.73	577
	Δt=5		2007	2012	0.67	700 %		1454
Cluster 4	Δt=2	35	2013	2015	0.33	300 %	23.20	217
	Δt=5		2013	2018	0.33	600 %		465
Cluster 5	Δt=2	32	2014	2016	0.67	300 %	13.19	111
	Δt=5		2011	2016	0.33	600 %		111

Note: Novelty, growth rate, coherence and scientific impact are defined in Section 2.2. Source: Own calculation based on the dataset.

We take cluster 1 (Technology Topic Analysis) as an example to elaborate on the meaning of the data in the table. When the time interval parameter is set to 2, cluster 1 has the highest growth rate from 2015 to 2017, which is 433 %. There is 1 smoothed number of publications in the first year (2015) when this topic began to gain popularity. In terms of scientific influence, the citation quantity of publications between 2015 and 2017 is 386. On the other hand, when the time interval parameter is set to 5, the growth rate from 2013 to 2018 is as high as 1800 %. When the topic began to increase, there are only 0.3 smoothed number of publications, and the scientific influence increased to 588. In addition, the consistency index of this cluster is good, indicating that it is a closely related topic. Therefore, regardless of the time interval parameter used, cluster 1 has been detected as an emerging research topic.

In contrast, cluster 2 (Technology Roadmapping) has the lowest growth rate among the five topics and the largest smoothed number of publications, regardless of the time interval parameter. Although the coherence and scientific impact (Δt=5) of this topic are excellent, it cannot be considered an emerging research topic because it does not satisfy the other attributes of the emerging topic. Similarly, cluster 4 (Technology Opportunity Analysis) and cluster 5 (Technology Management and Decision Support) satisfy the emergence attribute, and it is reasonable to consider them as emerging topics. Although cluster 3 (Technology Component Analysis) can be considered an emerging topic at the long-term time interval (Δt=5), its maximum growth rate occurred between 2007 and 2012, which is a relatively remote period, so we do not consider it an emerging topic. Moreover, based on the information in Fig. 6, we can see that the number of publications in clusters 2 and 3 has steadily increased over the long term without significant fluctuations. As a result, we conceive that these two topics are currently relatively mature.

Next, we will focus on considering the growth rate and novelty of three emerging research topics in the case of Δt=2. From Table 3, it can be seen that the earliest starting year for emerging topics was 2013. Therefore, starting in 2013, we calculated their growth rates and novelty separately. The relevant statistical data are shown in Table 4, Table 5. From the perspective of growth rate indicators, the growth rate of technology topic analysis is the most significant. Except for 2020, the growth rate of other years in this topic is greater than that of other research topics. The novelty indicator requires a relatively small number of publications at the beginning of a research topic, so this article regards the first half of the investigation period (2015–2018) as the early stage of emergence. From the statistical data in Table 5, it can be seen that technology opportunity analysis has obvious radical novelty. The number of publications on this topic is relatively small in the early stages, but it has developed rapidly since then, with the smoothed number of publications surpassing that of technology management and decision support.

**Table 4:** The growth rate on the research topic of TM (Δt=2).

Growth rate ($r_{i,t+\Delta t}$)	2017	2018	2019	2020	2021	2022
Cluster 1	433 %	257 %	162 %	133 %	210 %	233 %
Cluster 4	200 %	120 %	150 %	183 %	156 %	182 %
Cluster 5	150 %	100 %	100 %	133 %	144 %	188 %

Note: Growth rate is defined by equation (3) in Section 2.2.

**Table 5:** The novelty on the research topic of TM.

Novelty (${\bar{p}}_{i,t}$)	2015	2016	2017	2018	2019	2020	2021	2022
Cluster 1	1	2.33	4.33	6	7	8	14.67	18.67
Cluster 4	1	1.67	2	2	3	3.67	4.67	6.67
Cluster 5	2	2	3	2	3	2.67	4.33	5

Note: Novelty is defined by equation (2) in Section 2.2.

In summary, clusters 1, 4, and 5 are indeed radically novel, relatively rapidly growing, and coherent themes, and they have also had a major scientific impact. Technology roadmapping and technology component analysis are relatively mature research topics in tech mining. Furthermore, from the perspective of growth rate, technology topic analysis is a hot topic among emerging research topics. From the perspective of novelty indicators, technology opportunity analysis is a star topic among emerging research topics.

## 4 Discussion

This article provides a bibliometric literature review of tech mining literature in the past two decades, revealing the research foundation, development frontiers, and emerging developments in the field of tech mining through co-citation analysis and bibliographic coupling. Firstly, our review provides a detailed analysis of the foundation of tech mining research for the first time. Based on the results of co-citation analysis, we find that previous studies have mostly utilized text-based approaches and tools to analyze data to obtain technological intelligence such as trend analysis, technology forecast, R&D management, and so on. This is consistent with the results of previous narrative reviews [5,6]. On the other hand, compared with previous research results, our analysis results show that tech mining research is mainly rooted in bibliometrics and selectively based on patent analysis. Moreover, our analysis results emphasize that tech mining literature often provides practical insights for organizational strategic technology management and planning from the perspective of technological information.

Secondly, the bibliographic coupling results of this article validate the existing research viewpoint that the field of tech mining increasingly emphasizes the development and application of advanced tech mining indicators and technology prediction measures [7,118]. Based on bibliographic coupling results, our analysis indicates that researchers have recently not only focused on patent databases but also increasingly focused on scientific publications and other new types of data, such as online data and social media data, to maximize the potential of various ST&I data. Furthermore, it has become a research trend of tech mining to transform the mined technological intelligence into practical applications of technology innovation management, such as technology roadmapping, technology opportunity analysis, and technology management and decision support. On the other hand, research on technology topics and technology components has enriched the research results of existing tech mining reviews at the micro level, and technology topic research is an emerging research model. Technology component research has a high citation influence, and these two types of research have enlightening effects on future research.

Finally, this article identifies emerging research topics in the development frontiers of technology mining. Technology topic analysis, technology opportunity analysis, and technology management and decision support are emerging topics in this field. Specifically, technology topic analysis is a hot topic among the emerging research topics of technology mining, and technology opportunity analysis is a star topic among them. These two research topics are fertile ground for technology mining research, and more resources need to be invested to enrich these two types of research, in order to facilitate and strengthen the development of technology mining and promote scientific and technological progress.

## 5 Conclusions

Although the research results of this article contribute to familiarizing and understanding the field of tech mining, our research has some limitations. Firstly, the tech mining dataset in this article only includes scientific publications related to tech mining in the titles, abstracts, and keywords from the WOS database. We did not consider other databases such as Scopus, Science Direct, Springer Link, etc. This may reduce the recall of our dataset. Secondly, when exploring the foundation of tech mining research, this article sets the minimum citation threshold for references in co-citation analysis to 8. This means that we may overlook recently published articles that have rapidly increased citation influence, which may reduce the citation time for certain cluster themes. Finally, citation-based bibliometric methods may overstate the degree of correlation between publications, leading to biased results [[119], [120], [121], [122]].

Although this article has the aforementioned limitations, our research findings have implications for future research directions in the field of tech mining. First, the co-citation analysis and bibliographic coupling results show that patents and scientific publications are important data sources for tech mining research. However, combined with the manual coding, we find that few articles consider both patents and publications, and there are even fewer studies exploring the relationship between the two to obtain technological intelligence. In the process of transforming scientific research achievements into commercial applications, there will be a bottleneck for technological transformation due to various reasons, also known as the ‘Valley of Death’ [123,124]. Although the bottleneck of technological transformation is an obstacle, it is also a potential technological opportunity to some extent. Once the bottleneck is broken, it will bring huge value. Exploring and identifying the bottleneck of scientific and technological transformation needs to focus on the connection zone between science and technology, so it is particularly necessary to execute conjoint analysis in combination with scientific publications and patents. Therefore, this indicates the research potential and further research direction of patents and scientific publications.

Secondly, according to the cluster content analysis of bibliographic coupling, we find that the research on technology roadmapping has always been the focus of this research field. With the development of tech mining techniques, more and more research and application of machine learning, neural networks, link prediction, and other methods to automatically generate technology roadmapping. In addition, the patent map is an effective means to discover potential technological opportunities. To improve the application effect of patent maps in practice, scholars have continuously improved patent maps through keywords, local outlier factor, and other methods [96,115]. The technology opportunity landscape map proposed by Jeong et al. [125] is also an effective tool for analyzing technological opportunities. Therefore, developing and applying visualization methods to analyze text data and obtain useful intelligence is one of the key research directions in tech mining.

Thirdly, from the emerging topic detection results, we can see that technology topic analysis, technology opportunity analysis, and technology management and decision support are emerging research topics in Technology Minin (10.13039/501100010693TM). Technology topic analysis is growing rapidly and plays a dominant role in TM. Most studies identify potential technical topics through LDA topic modeling and/or other methods and conduct more in-depth TM research on this basis. On the other hand, technology opportunity analysis is a very novel research topic with great research potential, aiming to discover potential and new technology opportunities. These two topics have been applied in many emerging technological fields. Therefore, utilizing tech mining to analyze technological topics in emerging technology fields and identify technological opportunities and threats is one of the future research hotspots.

Finally, since the term ‘technology mining’ was proposed, most research topics have been of concern to researchers. Although this long-term research and investigation can provide us with more comprehensive knowledge and insights in the field of tech mining, there is also a lack of new and valuable research topics to drive the continuous development of tech mining research. Consequently, our research findings suggest the need to search for broader theories and concepts for tech mining research to expand its breadth and depth. We hope that this study can motivate scholars to engage in such work.

## Data availability statement

Data used in this article can be found in article/supp. material/reference in article.

## CRediT authorship contribution statement

**Xinyue Hu:** Writing – review & editing, Writing – original draft, Methodology, Formal analysis, Conceptualization. **Huiming Gu:** Writing – review & editing, Writing – original draft, Methodology, Formal analysis, Conceptualization. **Yongli Tang:** Writing – review & editing, Writing – original draft, Methodology, Formal analysis, Conceptualization. **Bo Wang:** Writing – review & editing, Writing – original draft, Methodology, Formal analysis, Conceptualization.

## Declaration of competing interest

The authors declare the following financial interests/personal relationships which may be considered as potential competing interests:Xinyue Hu reports financial support was provided by National Social Science Fund of China.

## Footnotes

1 Keyword analysis results are not reported here for brevity but can be made available from the corresponding author upon request.

2 Similarly, keyword analysis results are not reported here for brevity but can be made available from the corresponding author upon request.
