# Supplementary material for: Mapping the field: A bibliometric literature review on technology mining
Source: Heliyon. 2023 Dec 14;10(1):e23458. doi: 10.1016/j.heliyon.2023.e23458 (PMC10767374; doi:10.1016/j.heliyon.2023.e23458)
Supplement: Multimedia component 2 [file mmc2.docx]

Supplementary Material

Supplementary Material: Supplementary Material- Manual Coding.xlsx.


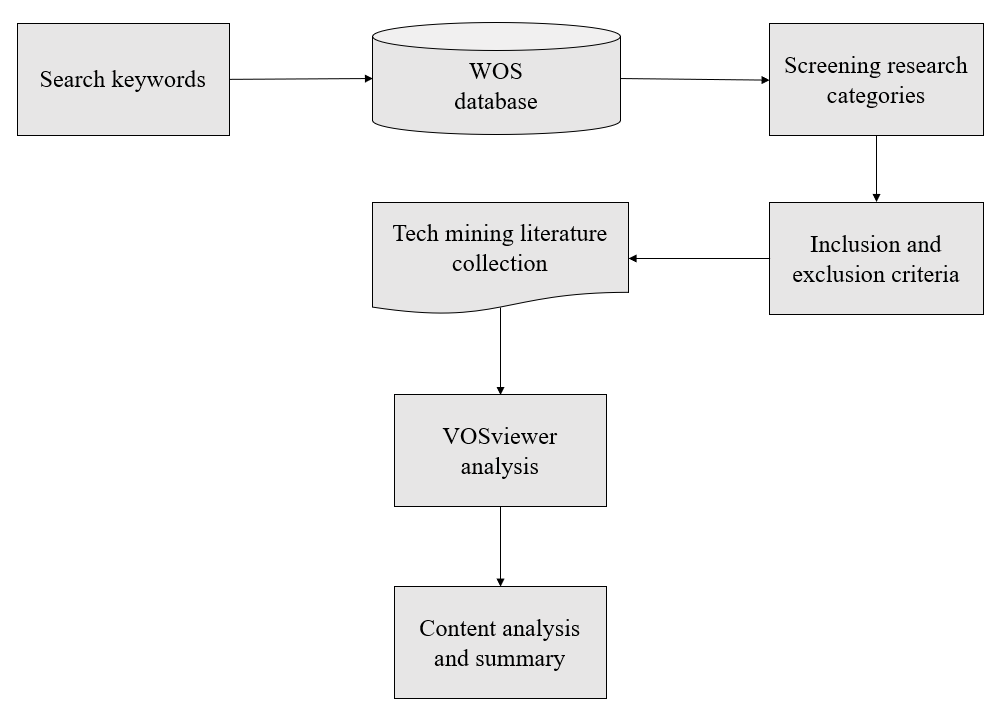


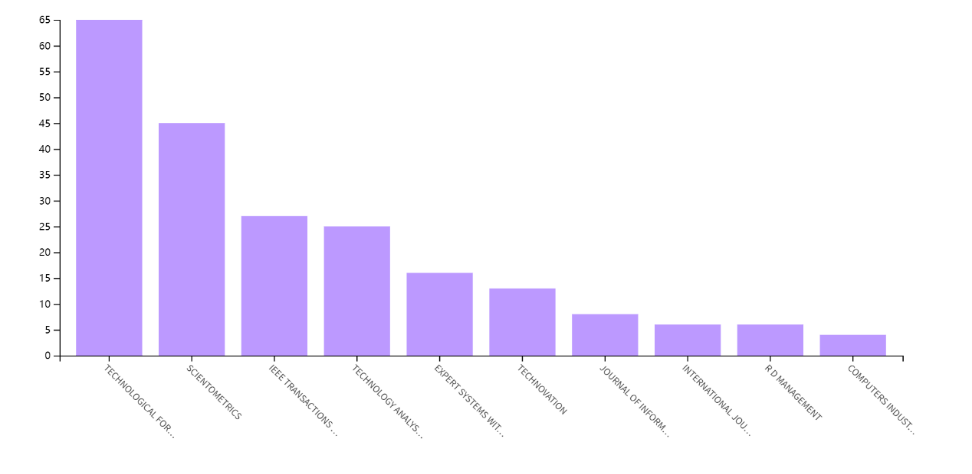
Figure 1. Overall research framework


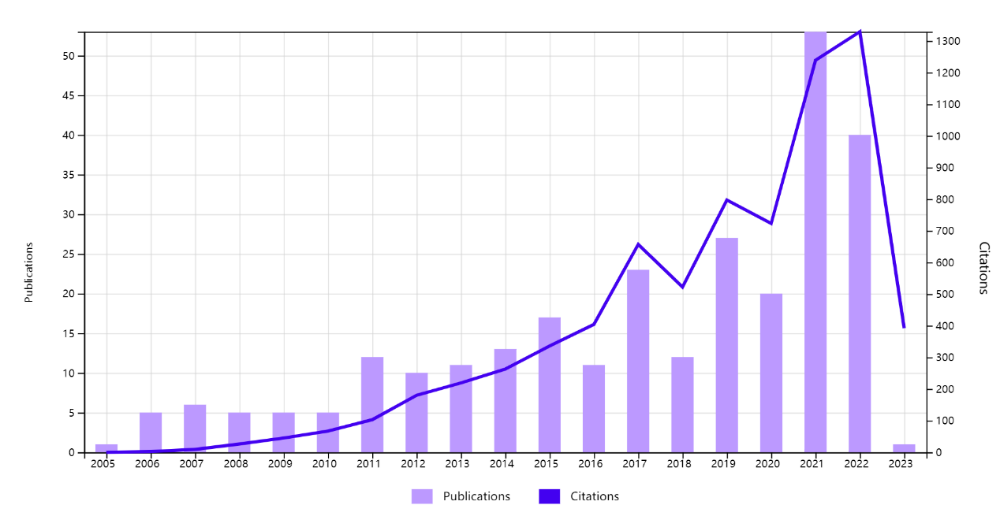
Figure 2. Distribution of source publication names

Figure 3. Citation frequency and publication distribution by year


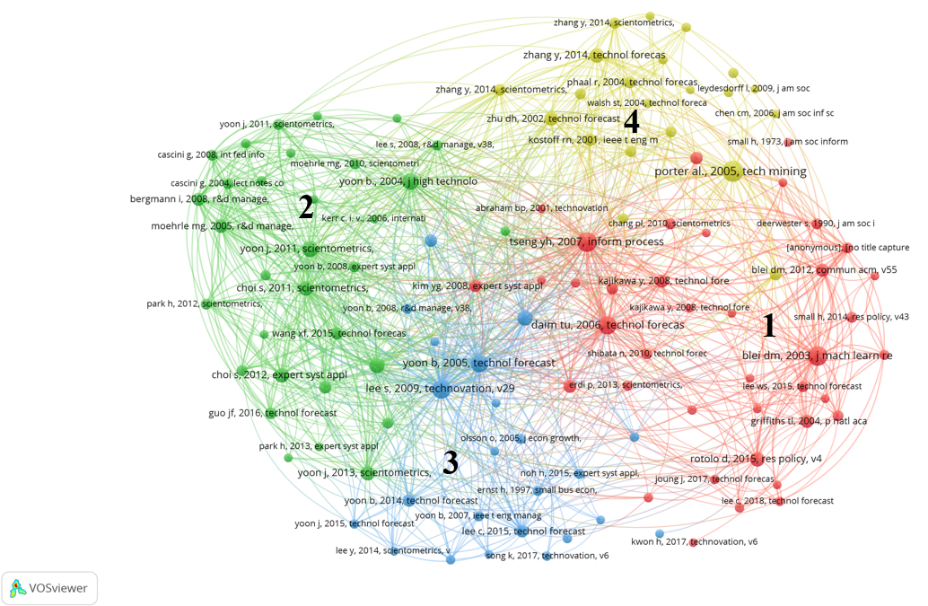


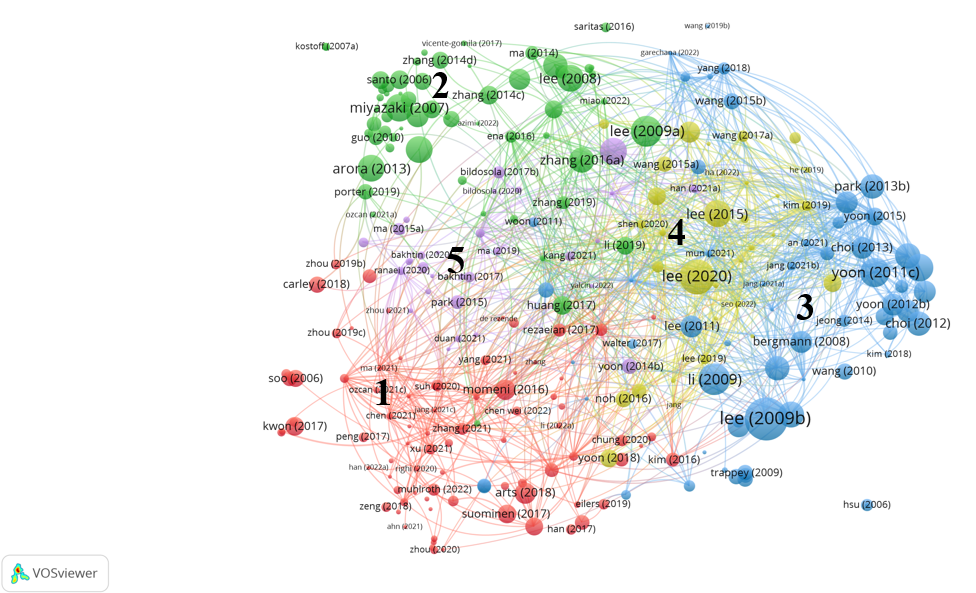
Figure 4. Co-citation network of the references cited by publications on tech mining


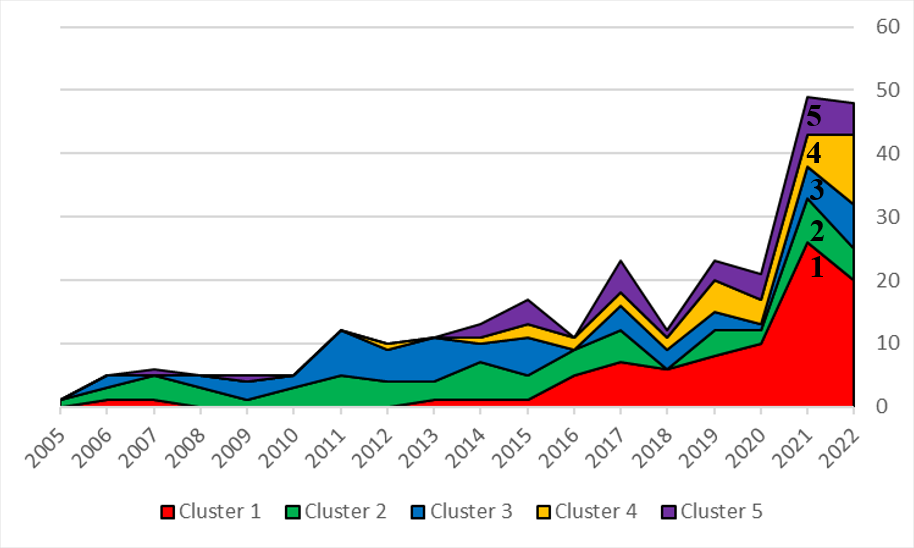
Figure 5. Bibliographic network of science publications on TM based on bibliographic coupling

Figure 6. Distribution of the number of published papers per cluster per year
